# Sporadic Renal Hemangioblastoma: A Case Report of a Rare Entity

**DOI:** 10.7759/cureus.47102

**Published:** 2023-10-16

**Authors:** Fnu Raja, Vinesh Kumar, Azzam Hammad, Caroline Abramovich

**Affiliations:** 1 Pathology, MetroHealth Medical Center, Cleveland, USA

**Keywords:** angiomyolipoma, renal cell carcinoma, immunohistochemistry, von hippel‐lindau disease, renal hemangioblastoma

## Abstract

Hemangioblastoma, also known as capillary hemangioblastoma, is a rare benign mesenchymal tumor commonly found in the central nervous system (CNS). It can also manifest in various organs, including the kidney. Renal hemangioblastoma (RH) is often associated with Von Hippel‐Lindau (VHL) disease, but sporadic occurrences are observed infrequently. While RH is usually asymptomatic, it can also cause abdominal pain and hematuria. In this study, we present a case of an elderly patient without history of VHL but complaining of abdominal pain for three days. Serological evaluations were unremarkable, and a CT scan identified a 2.4 cm mixed solid-cystic mass lesion on the left kidney's superior aspect. The patient subsequently underwent a biopsy followed by lesion ablation. Microscopic analysis revealed sheets of eosinophilic cells with ovoid nuclei, showing focal rhabdoid and spindle cell features, with an intricate capillary network. Focal nuclear atypia without necrosis or mitosis was noted. Immunohistochemistry (IHC) demonstrated positive staining for inhibin, S100, PAX8, and vimentin, along with patchy positivity for CD10 and RCC. Negative staining was observed for cytokeratin AE1/AE3, CK7, EMA, CK8/18, desmin, and HMB-45. The overall morphological characteristics and distinct IHC markers were consistent with RH. Although its pathogenesis remains unclear because of its rarity, distinguishing RH from renal cell carcinoma is crucial. IHC markers facilitate differentiation among lesions. The preferred treatment involves ablation or partial nephrectomy. Further assessment for possible VHL syndrome is essential, considering the distinct management approaches for sporadic and VHL-linked RH.

## Introduction

Hemangioblastoma represents one of the rarest benign mesenchymal tumors. It predominantly occurs in the central nervous system (CNS), particularly in the cerebellum [1]. It is also known as capillary hemangioblastoma and is characterized by slow growth [2]. Roughly 25% of these tumors are associated with Von Hippel‐Lindau (VHL) disease [3]. Apart from the CNS, hemangioblastoma can also manifest in other organs of the body, including the skin, liver, pancreas, lungs, urinary bladder, and kidney. In the Kidney, it is commonly linked to VHL, although sporadic cases are infrequently observed [4]. In terms of age distribution, renal hemangioblastoma (RH) exhibits a wide range, occurring between 16 and 71 years, with an equal distribution between males and females. This contrasts with CNS hemangioblastoma, which predominantly affects males [4]. Clinically, RH is usually asymptomatic, but in certain instances, it can cause nonspecific abdominal pain and hematuria.

VHL disease is a hereditary tumor syndrome inherited in an autosomal dominant pattern, occurring due to mutations in the VHL tumor suppressor gene located on the short arm of chromosome 3. The presence of non-functional VHL protein plays a significant role in the initiation of tumor growth. This disorder is characterized by the formation of both benign and malignant tumors in various parts of the body, such as the CNS, kidney, adrenal gland, and pancreas [5].

RH occurring sporadically without VHL disease is an infrequent event. Nevertheless, it exhibits a similar morphology to that observed in CNS hemangioblastoma. Under the microscope, both lesions display oval to polygonal cells with eosinophilic cytoplasm. Additionally, there is a notable presence of extensive vascularity characterized by thin to thick-walled blood vessels [6].

In terms of its diagnosis, there is no specific serological marker, and levels of erythropoietin (EPO) typically remain within the normal range, with polycythemia being a rare occurrence [7]. Radiological investigations may reveal a non-specific mass lesion, lacking distinct specificity. Usually, it presents as a unilateral solid mass, although in rare instances, some cystic lesions can also be observed [4].

To the best of our knowledge, the literature contains records of approximately 26 reported cases of sporadic RH [8]. In this case study, we present a case of an elderly patient who presented with non-specific abdominal pain and had no history of VHL disease. Alongside this case presentation, we provide a brief review of existing literature to gain better insights into the clinical and pathological characteristics of sporadic RH. Additionally, we aimed to distinguish this condition from other closely related renal tumors to improve our understanding of its unique features.

## Case presentation

**Figure 1:**
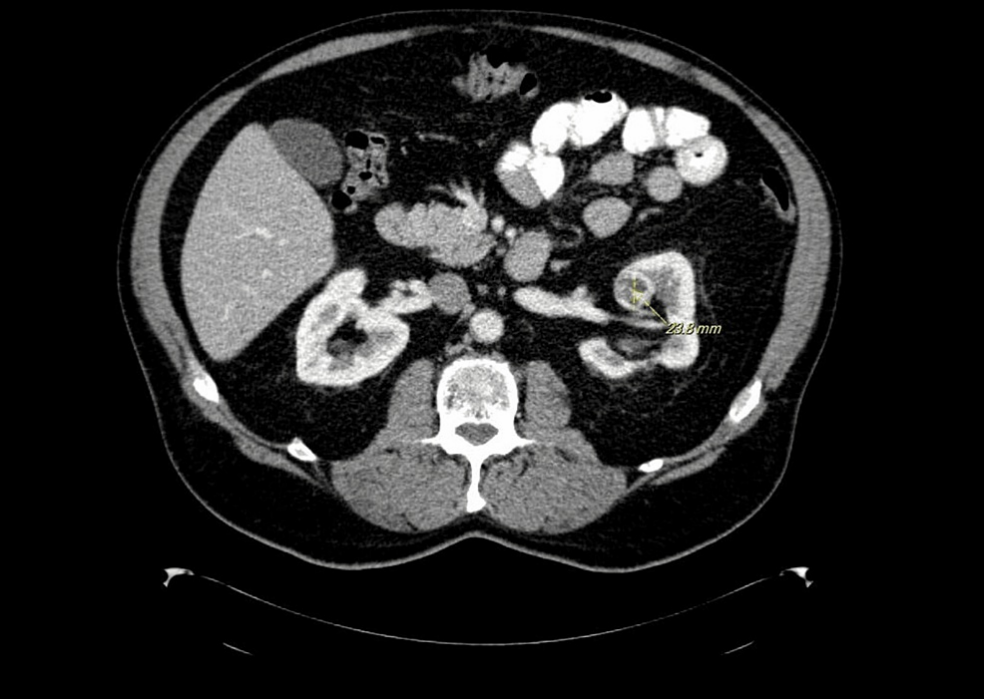
CT-scan revealed a mixed solid and cystic mass lesion on the superior pole of the left kidney.

An elderly male patient with a past medical history of chronic hepatitis C, diverticulitis, and GERD presented to the outpatient clinic with complaints of nonspecific left abdominal pain for three days. The patient described the pain as moderate in intensity, non-radiating with no aggregating or relieving factors. The patient denied vomiting, diarrhea, constipation, fevers, shortness of breath, or any other associated symptoms. Physical examination revealed mild to moderate pain on palpation; however, no distinctive mass was found. The serologic workup was unremarkable. An abdominopelvic CT scan revealed a 2.4 cm mixed solid-cystic mass lesion located on the superior pole of the left kidney (Figure 1), suspicious of malignancy. However, there was no evidence of renal vein invasion or metastasis. The patient underwent a needle core biopsy and ablation of the lesion.

Grossly, the specimen consisted of three tan needle core biopsies ranging from 0.2 to 1.2 cm in length and 0.1 cm in diameter.

**Figure 2:**
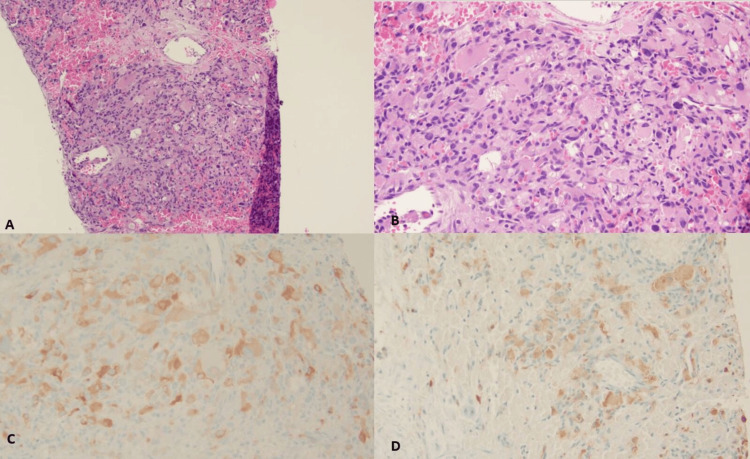
Low-power 4x shows a solid lesion with arborizing thin-walled vessels on H&E (A). Solid lesion shows cells with an abundant amount of eosinophilic cytoplasm, ovoid nuclei, and focal rhabdoid features on H&E (B). Eosinophilic cells were positive for inhibin (C) and S100 (D).

A histopathologic examination of the lesion revealed that the tumor was composed of sheets of eosinophilic cells with ovoid nuclei, showing focal rhabdoid and spindle cell features, haphazardly arranged with a delicate, arborizing capillary network. Focal nuclear atypia was identified; however, no necrosis or mitosis was identified (Figures 2A-2B). For further characterization of the lesion, immunohistochemistry (IHC) was done, which revealed the eosinophilic cells to be positive for inhibin and S100 (Figures 2C-2D), PAX8, and vimentin. CD10 and RCC showed patchy positive reactions, while the cells were negative for cytokeratin AE1/AE3, CK7, EMA, CK8/18, desmin, and HMB-45. Background histocytes/macrophages were highlighted by CD68 and CD163, and CD31 highlighted the endothelial cells.

The morphologic features, along with the unique immunophenotype, were consistent with renal hemangioblastoma. Upon one-month follow-up, the patient did not show any signs or symptoms of recurrence.

## Discussion

A sporadic form of RH is rare and of unknown pathogenesis/etiology. As only a few cases have been reported in the literature, its progression and reoccurrence rate are still not well-defined. Currently, there is no specific molecular test available for this entity. In the kidney, RH may resemble other primary benign and malignant lesions. It is challenging to diagnose RH based on clinical-radiologic features alone. Microscopically, the histological features may overlap with other lesions, especially clear cells, renal cell carcinoma (RCC), and epithelioid variants of angiomyolipoma, especially on a core needle biopsy [9]; however, it is very important to differentiate these lesions from one another because of different management plans and different prognoses.

As in our case, both RCC and RH can show eosinophilic cells with focal rhabdoid and spindle cells and haphazardly arranged delicate and arborizing capillary networks, and distinguishing between these two entities morphologically can be extremely challenging on a small needle biopsy. Moreover, both lesions can show solid sheets or a nested pattern. However, these two lesions may be differentiated based on their distinctive IHC markers. Almost all cases of RH show strong positivity for α-inhibin, S100, and NSE, unlike RCC. Furthermore, RCC shows positivity for AE1/AE3, EMA, PAX8, PAX2, CD10, and RCC [10].

Another differential consideration includes the epithelioid variant of angiomyolipoma. Both lesions show cells with clear or eosinophilic cytoplasm, accompanied by an extensive capillary network. Nonetheless, angiomyolipoma additionally encompasses smooth muscle and adipose tissue components, which are not characteristic of RH. The differentiation between these two entities is adequately achieved through IHC. This allows for the definitive exclusion of angiomyolipoma. Notably, angiomyolipoma displays positive expression for HMB45 while being negative for α-inhibin and S100. This specific immune profile further aids in excluding angiomyolipoma as a diagnosis [10].

Treatment for RH involves surgical removal of the lesion, either by ablation or partial nephrectomy [11].

## Conclusions

RH is a benign entity that carries an excellent prognosis. Clinical presentation along with radiographic features is not sufficient for the diagnosis. Careful histopathologic examinations along with the proper use of IHC markers are mandatory to finalize the diagnosis and prevent the patient from overtreatment. Although sporadic cases are rarely reported compared to lesions arising in association with VHL syndrome, it is highly recommended to further evaluate the patient for the possibility of VHL syndrome, as sporadic and VHL-linked RH carry different management plans.
